# Nonlinear compartmental modeling of COVID-19 with dual dose vaccination using Mason graphs and variational iteration method

**DOI:** 10.1038/s41598-025-34692-y

**Published:** 2026-01-06

**Authors:** Umer Ghani, Bilal Ahmad, Shahid Mahmood, Aymen Flah, Mohammad Ghatasheh, Ivo Pergl

**Affiliations:** 1 Department of Mathematics, University of Wah (UOW), Wah Cantt, 47040 Pakistan; 2 Department of Mathematics, Government Degree College, KDA Township, Kohat, Pakistan; 3 Applied Sciences Research Centre, Applied Sciences Private University, Amman, 11931 Jordan; 4 ENET Centre, CEET, VSB-Technical University of Ostrava, 70800 Ostrava, Czech Republic; 5 College of Engineering, University of Business and Technology, Jeddah, 21448 Saudi Arabia; 6 Department of Basic Sciences, Middle East University, Amman, 11831 Jordan

**Keywords:** Model, SEQIR model, SIR model, SEIR model, Variational Iteration Method (VIM), Mason Graph, National Command and Operation Centre (NCOC), Computational biology and bioinformatics, Diseases, Mathematics and computing

## Abstract

In this research work, a novel non-linear mathematical model has been proposed considering susceptible, quarantined, infected, recovered, and removed compartments before and after the 1st dose and 2nd dose of vaccination. For this dynamics model, the novel coronavirus COVID-19, a contagious disease, is taken as a case study in which its transmission, impact of vaccination, and mitigation have been discussed. This model may be helpful in numerous fields of epidemiology and dynamical systems; moreover, Mason Graph has been used to describe the mathematical model. The stability analysis and disease-free equilibrium points have been deliberated for the model. In this work, the semi-analytical technique Variational Iteration Method has been employed, which will assist researchers in the future by showing that if the rate of immunized personnel rises, then the infection rate decreases. It has been observed that the non-vaccinated personnel decrease with the passage of time due to the awareness campaign programs of the governments. Furthermore, it was observed that the removed rate also decreases with the passage of time as the immunized personnel rises. Mathematical software MAPLE has been used to calculate the analytical solutions of the aforementioned mathematical model.

## Introduction

In the late 1960s, virologists conducted vigorous studies on a number of human strains and animal viruses, such as infectious bronchitis virus, mouse hepatitis virus and transmissible gastroenteritis virus of swine. The research which was started from the common cold has now reached the investigation of coronaviruses. The recent COVID-19 pandemic has been an exceptional calamity in healthcare, economic development and social life. The emergency nature of the situation is fortified by findings that the virus is constantly mutating and has the potential of becoming highly pathogenic in humans. Epidemiological studies of coronaviruses reveal that the novel virus causes a variety of respiratory diseases such as pneumonia, chronic bronchitis and asthma being predominant illnesses in young adults, elders, and children in 2005 history by Kahn^1^. It has also been observed that different animal species such as mice, rats, cats, dogs, turkeys, chickens, pigs, and rabbits are the possible reservoirs of coronaviruses^2^.

From the literature, it has been noticed that in many cases these viruses may share:

1. similar symptoms, including cough, runny nose, sore throat, fever, headache, and fatigue; they spread in similar ways.
2. the occurrence rate of this novel virus is more rapid in spring, winter, and rainy seasons compared to summer seasons^3^.
3. the helicase amino acid sequence is 50%–88% similar among beta-coronaviruses^4^.
4. the basic genomic structure and replication cycle of SARS-CoV-2 are similar to those of other coronaviruses.
5. the main functions of their structural and nonstructural proteins are also conserved.
6. the immune pathogens of severe COVID-19 is similar to that of SARS and MERS, which involves excessive cytokines release^5^. Hence, it is a coronavirus like other types of coronaviruses^6^.

**Figure 1:**
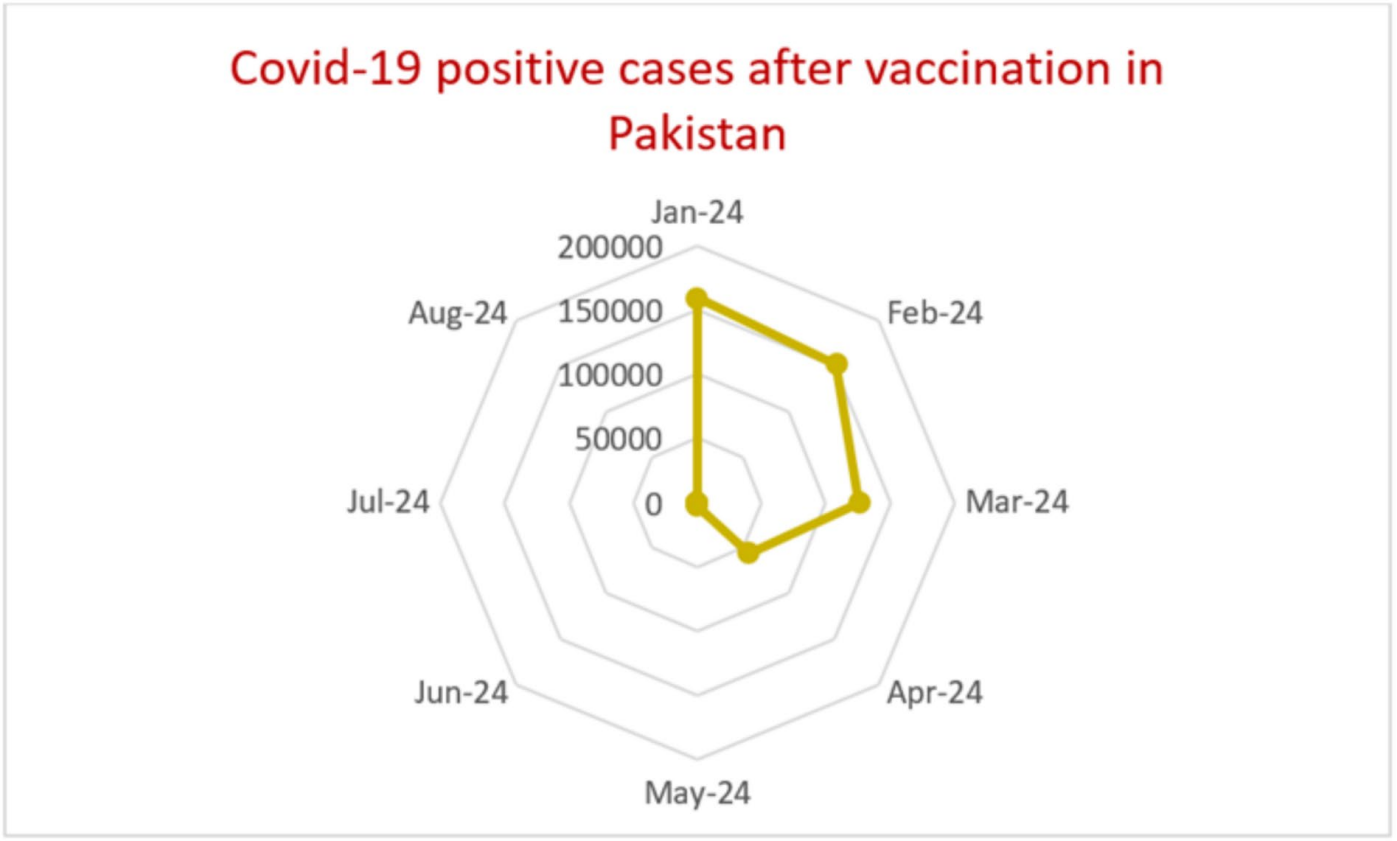
Statistical trends of COVID-19 positive cases in Pakistan after the rollout of vaccination.

The World Health Organization (WHO) database confirms that, as of 20th March 2024, 201 countries affected by COVID-19 reported 704,753,890 confirmed cases and 7,010,681 deaths^7^. However, the infection and death rates change every second^8^. After proper vaccination, the infection rate has decreased^9^, as shown in Figure 1.

Through the recent COVID-19 experience, professionals are developing new mathematical models to analyze and elaborate the diffusion of epidemiological diseases, similar to those developed for SIR, SEQIR, and SEIR models. This type of mathematical modeling plays an important role in estimating the number of worst- and best-case scenarios when the transmission dynamics of the epidemiological disease are unknown^10^. It also helps to estimate the precautionary measures adopted against the novel coronavirus, such as quarantining, social distancing, and vaccination.

All preventive techniques aim to keep the basic reproduction number less than 1, whereas diminution strategies aim to reduce the effect of the outbreak. Recently, researchers have used mathematical models to investigate the impact of control measures on the spread of COVID-19 in human populations. The aim of this research work is to investigate an improved SEQIR COVID-19 compartmental mathematical model by introducing the 1st and 2nd doses of vaccination and a removed compartment to simulate COVID prevalence. The authors elaborate important aspects of the COVID-19 outbreak: i) how the transmission of COVID-19 is reduced after double-dose vaccination, ii) how the overall death rate is affected by COVID-19 including natural death. If the basic reproduction number is less than one (i.e., $R_0 < 1$), then the fatal disease will be eradicated; if the basic reproduction number is greater than one (i.e., $R_0 > 1$), then an epidemic will occur. These findings show how the rate of transmission, the rate of progression, and the rate of double-dose vaccination affect the dynamics of COVID pervasiveness.

In the present work, we propose a new epidemiological compartmental model based on the SEQIR model^11^ that describes the super-spreading phenomena of coronavirus and the decline in virus spreading after dual-dose vaccination. In this research, we use the Variational Iteration Method, a semi-analytical technique, to solve the novel COVID-19 model^12^. This technique provides a solution in a rapidly convergent series. The authors develop the correction functional and calculate Lagrange multipliers using this technique. The main advantages of this technique are: i) freedom from round-off errors, ii) no perturbation, iii) no restrictive assumptions or transformations, and iv) implementation without discretization. The analytical solution of the novel COVID-19 model was calculated using computational software such as MATLAB and MAPLE.

## Derivation of novel coronavirus disease model

This section presents a modified COVID-19 SEQIR model that reflects the existing circumstances in Pakistan and neighboring regions. The non-linear mathematical model classifies the total population of Pakistan into seven sub-compartments: susceptible *S*, quarantined *Q*, infected *I*, recovered *R*, removed *D*, 1st dose vaccinated $V_1$, and 2nd dose vaccinated $V_2$ (those who have received both the first and second doses). Here we assume that *N*(*t*) is the total population size of Pakistan, represented by

$$N(t) = S(t) + Q(t) + I(t) + R(t) + D(t) + V_1(t) + V_2(t).$$

**Figure 2:**
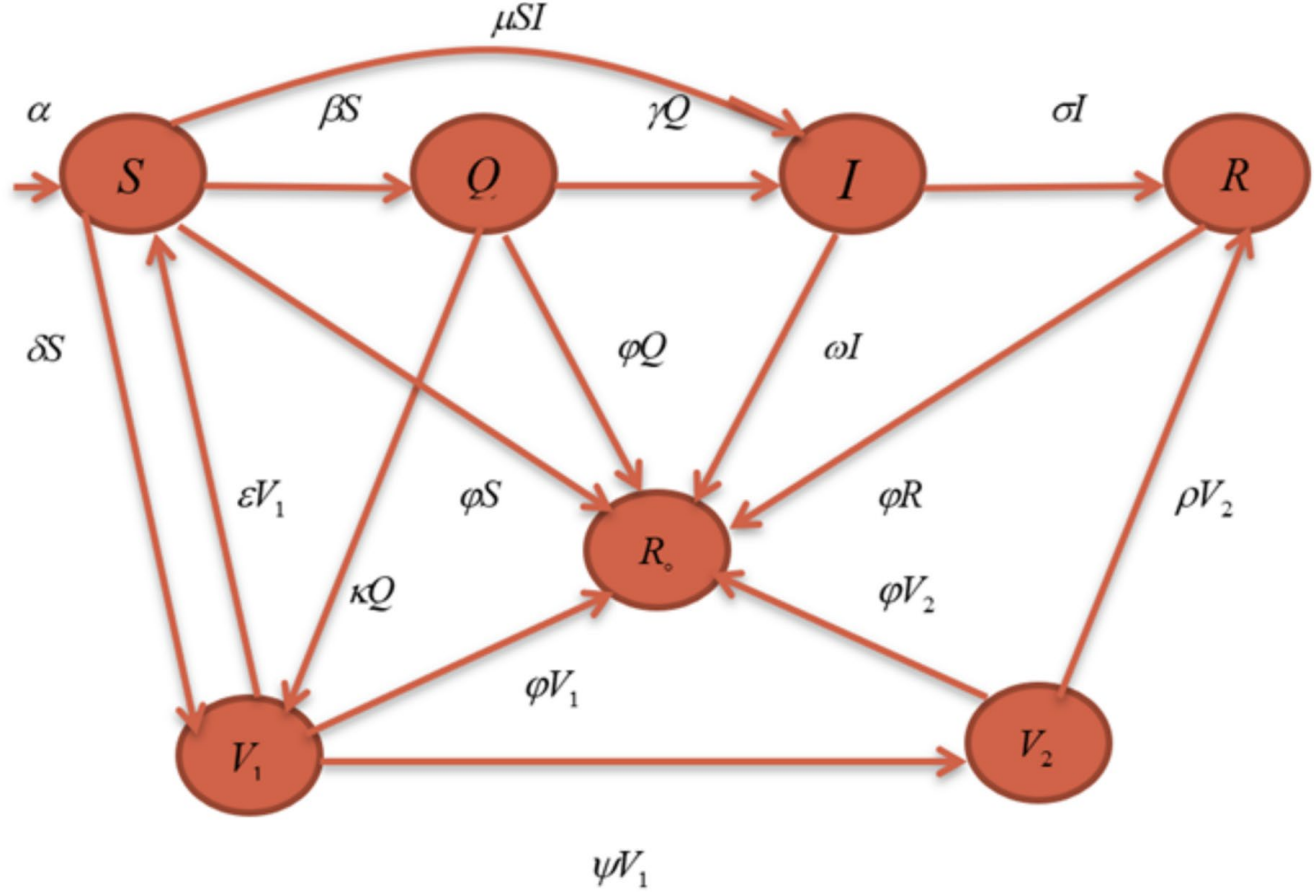
Signal flow graph illustrating the non-linear mathematical dynamics model.

In this non-linear mathematical model, the partition between the common population and susceptible or infected population is known as quarantine. The removed compartment includes those who died due to COVID-19 or any other reason in any compartment. *R* represents the population recovered from COVID-19 disease. Moreover, we include the total inflow of susceptible Pakistani population into the country at a rate $\Pi$ per unit time. In Figure 2, the flow diagram of the non-linear mathematical dynamics model is portrayed.

**Modeling of Susceptible population:** The susceptible population compartment is expanded by recruiting individuals at a rate $\Pi$ and is diminished by natural death rate $\mu$, quarantined rate $\beta$, vaccinated rate $\phi$, and infected rate $\lambda$. The susceptible population is amplified by the rate $\gamma _1$ as some individuals become susceptible again after receiving the first vaccine. The following differential equation governs the rate of change of the susceptible population:

1 $$\begin{aligned} \frac{dS}{dt} = \Pi - (\mu + \beta + \phi + \lambda )S + \gamma _1 V_1. \end{aligned}$$

**Modeling of Quarantine population:**The crucial incubation period for COVID-19 disease transmission is 4–14 days. To control transmission, infected individuals must be isolated from susceptible ones for 14 days. This isolated population is known as quarantined. The quarantined population is amplified at rate $\beta S$ from the susceptible population and diminished at rates $\phi _1$, $\mu$, and $\lambda _1$ due to vaccination, natural death, and infection. The differential equation is:

2 $$\begin{aligned} \frac{dQ}{dt} = \beta S - (\phi _1 + \mu + \lambda _1)Q. \end{aligned}$$

**Modeling of Infected population:** Individuals with confirmed positive COVID-19 reports or complete symptoms are infected. The infected population is amplified from susceptible at rate $\lambda S$ and from quarantine at rate $\lambda _1 Q$. It is diminished by recovery rate $\gamma$ and removed rate $\delta$. This stage is very dangerous as it may cause virus spreading. The differential equation is:

3 $$\begin{aligned} \frac{dI}{dt} = \lambda S + \lambda _1 Q - (\gamma + \delta + \mu )I. \end{aligned}$$

**Modeling of Recovered population:** We assume infected individuals recover from COVID-19 and those who received double-dose vaccination remain safe. The recovered compartment increases by recovery rate $\gamma I$ and safe rate $\gamma _2 V_2$ after double-dose vaccination, and decreases by natural death rate $\mu$. The differential equation is:

4 $$\begin{aligned} \frac{dR}{dt} = \gamma I + \gamma _2 V_2 - \mu R. \end{aligned}$$

**Modeling of Removed population:** The population that died due to any reason is included in the removed compartment. It increases with natural death rate $\mu$ and COVID-19 death rate $\delta$. The removed population only increases. The differential equation is:

5 $$\begin{aligned} \frac{dD}{dt} = \delta I + \mu (S + Q + I + V_1 + V_2). \end{aligned}$$

**Modeling of 1st dose vaccinated population:** Complete COVID-19 vaccination consists of two doses. After the 1st dose, the 2nd is given after 28 days. The 1st dose vaccinated population is amplified at rates $\phi S$ and $\phi _1 Q$ and decreased at rates $\gamma _1$ (back to susceptible), $\mu$ (natural death), and $\phi _2$ (to 2nd dose). The differential equation is:

6 $$\begin{aligned} \frac{dV_1}{dt} = \phi S + \phi _1 Q - (\gamma _1 + \mu + \phi _2)V_1. \end{aligned}$$

**Modeling of 2nd dose vaccinated population:** The 2nd dose vaccinated population increases at rate $\phi _2 V_1$ and decreases at natural death rate $\mu$ and recovery/safe rate $\gamma _2$. The differential equation is:

7 $$\begin{aligned} \frac{dV_2}{dt} = \phi _2 V_1 - (\mu + \gamma _2)V_2. \end{aligned}$$

The complete mathematical model is:

8 $$\begin{aligned} \begin{aligned} \frac{dS}{dt}&= \Pi - (\mu + \beta + \phi + \lambda )S + \gamma _1 V_1, \\ \frac{dQ}{dt}&= \beta S - (\phi _1 + \mu + \lambda _1)Q, \\ \frac{dI}{dt}&= \lambda S + \lambda _1 Q - (\gamma + \delta + \mu )I, \\ \frac{dR}{dt}&= \gamma I + \gamma _2 V_2 - \mu R, \\ \frac{dD}{dt}&= \delta I + \mu (S + Q + I + V_1 + V_2), \\ \frac{dV_1}{dt}&= \phi S + \phi _1 Q - (\gamma _1 + \mu + \phi _2)V_1, \\ \frac{dV_2}{dt}&= \phi _2 V_1 - (\mu + \gamma _2)V_2. \end{aligned} \end{aligned}$$

With initial conditions:

$$S(0) = S_0,\ Q(0) = Q_0,\ I(0) = I_0,\ R(0) = R_0,\ D(0) = D_0,\ V_1(0) = V_{10},\ V_2(0) = V_{20}.$$

All respective biological meanings and parameters are presented in Table 1.

**Table 1:** Elucidation of the parameters in the suggested dynamics model.

**Parameter**	**Denotation**
$\Pi$	The susceptible population growth rate
$\beta$	The rate from susceptible to quarantine
$\lambda$	The rate from susceptible to infected
$\lambda _1$	The rate from quarantined to infected
$\gamma$	The rate from infected to recovered
$\mu$	Natural death rate
$\delta$	COVID-19 death rate
$\phi$	The transmission rate from susceptible to 1st dose vaccinated
$\gamma _1$	The transmission rate from 1st dose vaccinated back to susceptible
$\phi _1$	The transmission rate from quarantined to 1st dose vaccinated
$\phi _2$	The transmission rate from 1st dose vaccinated to 2nd dose vaccinated
$\gamma _2$	The transmission rate from 2nd dose vaccinated to recovered

## Elementary characteristics

### Non-negativity of solution

#### Theorem 1

All solutions of the model with non-negative initial conditions remain non-negative for $t \ge 0$.

#### Proof

Since the right-hand side of the mathematical dynamics model structure is completely continuous and locally Lipschitzian on *D*, the solution (*S*(*t*), *E*(*t*), *I*(*t*), *R*(*t*), *V*(*t*), *A*(*t*), *H*(*t*)) with initial conditions exists and is unique on $[0, \tau )$ where $\tau > 0$. From the aforesaid model structure we have, with initial conditions,

$$\dot{S} \ge 0 \quad \text {when} \quad S = 0.$$

Now for the remaining six equations of the above-mentioned model we have, with initial conditions,

$$\dot{E} \ge 0 \quad \text {when} \quad E = 0,$$

$$\dot{I} \ge 0 \quad \text {when} \quad I = 0,$$

$$\dot{R} \ge 0 \quad \text {when} \quad R = 0,$$

$$\dot{V} \ge 0 \quad \text {when} \quad V = 0,$$

$$\dot{A} \ge 0 \quad \text {when} \quad A = 0,$$

$$\dot{H} \ge 0 \quad \text {when} \quad H = 0,$$

where

$$\dot{E}\big |_{E=0} = \beta S I + \cdots \ge 0,$$

and similarly for the others (the exact expressions depend on the model terms, but all inflows are non-negative when other variables are non-negative). This completes the proof of the theorem because we have seen that,

$$S(t) \ge 0, \quad E(t) \ge 0, \quad I(t) \ge 0, \quad R(t) \ge 0, \quad V(t) \ge 0, \quad A(t) \ge 0, \quad H(t) \ge 0$$

$$\square$$

#### Invariant region

##### Theorem 2

All solutions are bounded and enter the region

$$\Omega = \left\{ (S,Q,I,R,D,V_1,V_2) \in \mathbb {R}^7_+ : N(t) \le \frac{\Pi }{\mu } \right\}$$

as $$t \rightarrow \infty$$.

##### Proof

Let (*S*(*t*), *E*(*t*), *I*(*t*), *R*(*t*), *V*(*t*), *A*(*t*), *H*(*t*)) be a solution of the model with non-negative initial conditions in $\Gamma$. Define the total population

$$N(t) = S(t) + E(t) + I(t) + R(t) + V(t) + A(t) + H(t).$$

Differentiating *N*(*t*) with respect to time *t*, we obtain

$$\frac{dN}{dt} = \dot{S} + \dot{E} + \dot{I} + \dot{R} + \dot{V} + \dot{A} + \dot{H}.$$

Substituting the expressions for $\dot{S}$, $\dot{E}$, $\dot{I}$, $\dot{R}$, $\dot{V}$, $\dot{A}$, and $\dot{H}$ from the model equations, we get

$$\frac{dN}{dt} = \Pi - \mu N - (\delta _I + \delta _A + \delta _H) \le \Pi - \mu N,$$

since mortality terms $(\delta _I, \delta _A, \delta _H) \ge 0$. Thus,

$$\frac{dN}{dt} \le \Pi - \mu N.$$

By the comparison theorem (or Gronwall’s inequality for differential inequalities), it follows that

$$N(t) \le z(t),$$

where *z*(*t*) solves the linear equation

$$\dot{z} = \Pi - \mu z, \quad z(0) = N(0).$$

The explicit solution is

$$z(t) = \frac{\Pi }{\mu } \left( 1 - e^{-\mu t}\right) + N(0) e^{-\mu t}.$$

As $$t \rightarrow \infty$$, $$z(t) \rightarrow \frac{\Pi }{\mu } = \Lambda$$. Therefore,

$$\limsup _{t \rightarrow \infty } N(t) \le \Lambda .$$

Combining this with the non-negativity established in Theorem 1, all solutions initiating in $\Gamma$ are bounded and ultimately enter the compact attracting region $\Omega$. This completes the proof. The above theorems on non-negativity and boundedness (invariant region) for the present non-linear mathematical dynamics model follow the standard approach initially developed for classical compartmental models such as the SEIR model and its extensions. $\square$

## Basic reproduction number and disease-free equilibrium

The Basic Reproduction Number, denoted $\mathscr {R}_0$, is defined as the expected number of secondary disease cases caused by introducing a single infective individual into a wholly susceptible population ^13^. The disease-free equilibrium (DFE) of the mathematical dynamics model is obtained by setting the derivatives to zero and assuming no infection, i.e., $E = I = A = H = R = V = 0$. At the DFE, we have

$$S^* = \frac{\Pi }{\mu }, \quad E^* = I^* = A^* = H^* = R^* = V^* = 0.$$

We employ the Next Generation Matrix Method ^14,15^ to derive the Basic Reproduction Number $\mathscr {R}_0$ for the model given by equations (9). The infected compartments are

$$E, \, I, \, A, \, H.$$

The rate of appearance of new infections $\mathscr {F}$ and the rate of transfer between infected compartments $\mathscr {V}$ are given by

9 $$\begin{aligned} \mathscr {F}&= \begin{pmatrix} \beta S I + \beta _q q \beta S (A + H) \\ 0 \\ 0 \\ 0 \end{pmatrix},&\mathscr {V}&= \begin{pmatrix} (\sigma + \mu ) E \\ -\sigma E + (\gamma _a + \gamma _I + \phi + \delta _I + \mu ) I \\ -\phi I + (\gamma _A + \delta _A + \mu ) A \\ -q \beta S (A + H) + (\rho + \delta _H + \mu ) H \end{pmatrix}. \end{aligned}$$

At the disease-free equilibrium, the Jacobian matrices of $\mathscr {F}$ and $\mathscr {V}$ with respect to the infected variables (*E*, *I*, *A*, *H*) are

10 $$\begin{aligned} F&= \begin{pmatrix} 0 & \beta S^* & \beta _q q \beta S^* & \beta _q q \beta S^* \\ 0 & 0 & 0 & 0 \\ 0 & 0 & 0 & 0 \\ 0 & 0 & 0 & 0 \end{pmatrix},&V&= \begin{pmatrix} \sigma + \mu & 0 & 0 & 0 \\ -\sigma & \gamma _a + \gamma _I + \phi + \delta _I + \mu & 0 & 0 \\ 0 & -\phi & \gamma _A + \delta _A + \mu & 0 \\ 0 & 0 & 0 & \rho + \delta _H + \mu \end{pmatrix}. \end{aligned}$$

The next generation matrix is $FV^{-1}$. The Basic Reproduction Number $\mathscr {R}_0$ is the spectral radius of $FV^{-1}$, which simplifies to

11 $$\begin{aligned} \mathscr {R}_0 = \frac{\beta \sigma S^*}{(\sigma + \mu )(\gamma _a + \gamma _I + \phi + \delta _I + \mu )}. \end{aligned}$$

Substituting $S^* = \frac{\Pi }{\mu }$, we obtain

12 $$\begin{aligned} \mathscr {R}_0 = \frac{\beta \sigma \Pi }{\mu (\sigma + \mu )(\gamma _a + \gamma _I + \phi + \delta _I + \mu )} = \mathscr {R}_I + \mathscr {R}_A + \mathscr {R}_H, \end{aligned}$$

where

$$\mathscr {R}_I = \frac{\beta \sigma \Pi }{\mu (\sigma + \mu )(\gamma _a + \gamma _I + \phi + \delta _I + \mu )}, \quad \mathscr {R}_A = q \beta _q \mathscr {R}_I \frac{\phi }{\gamma _A + \delta _A + \mu }, \quad \mathscr {R}_H = q \beta _q \mathscr {R}_I \frac{\rho }{\rho + \delta _H + \mu }.$$

Here, $\mathscr {R}_I$ represents the contribution from symptomatic infectious individuals, $\mathscr {R}_A$ from asymptomatic severe cases, and $\mathscr {R}_H$ from hospitalized cases. Thus, the Basic Reproduction Number of the model is

13 $$\begin{aligned} \mathscr {R}_0 = \frac{\beta \sigma \Pi }{\mu (\sigma + \mu )(\gamma _a + \gamma _I + \phi + \delta _I + \mu )}. \end{aligned}$$

This expression quantifies the potential for disease spread in the population and will be used in the subsequent stability analysis of the equilibria.

## Variational iteration method

**Figure 3:**
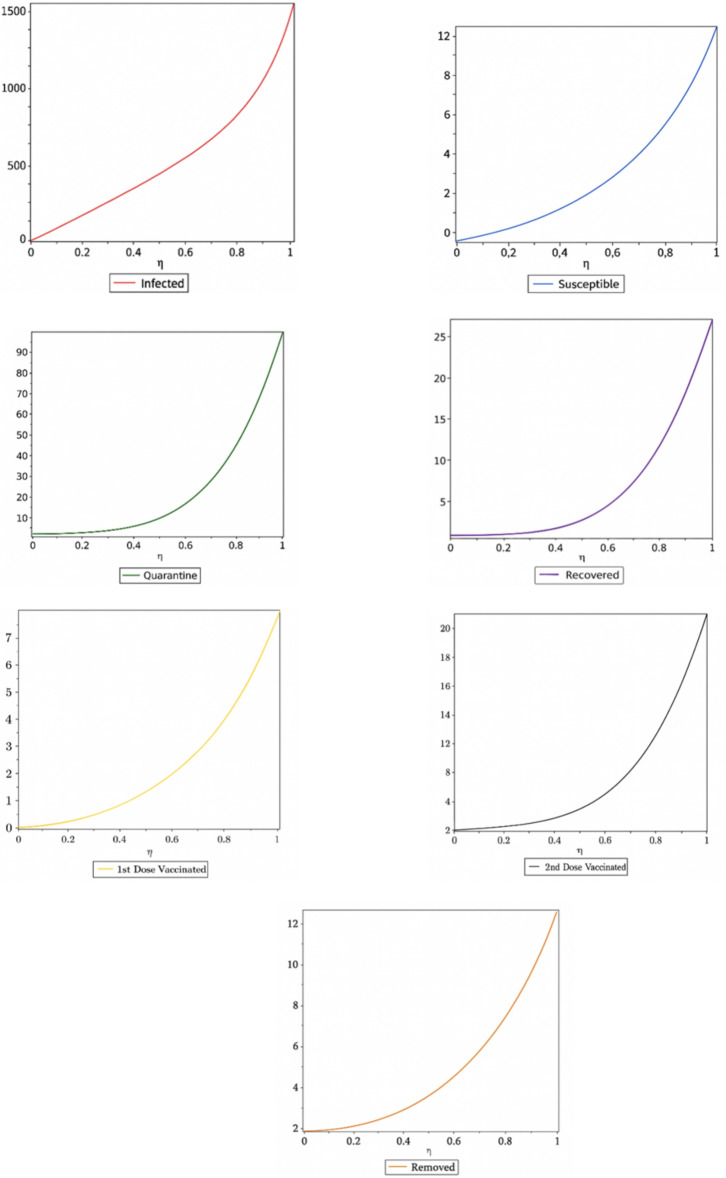
Population dynamics of susceptible, infected, quarantined, recovered, and removed individuals before vaccination, and after the first and second vaccination doses in Pakistan.

**Figure 4:**
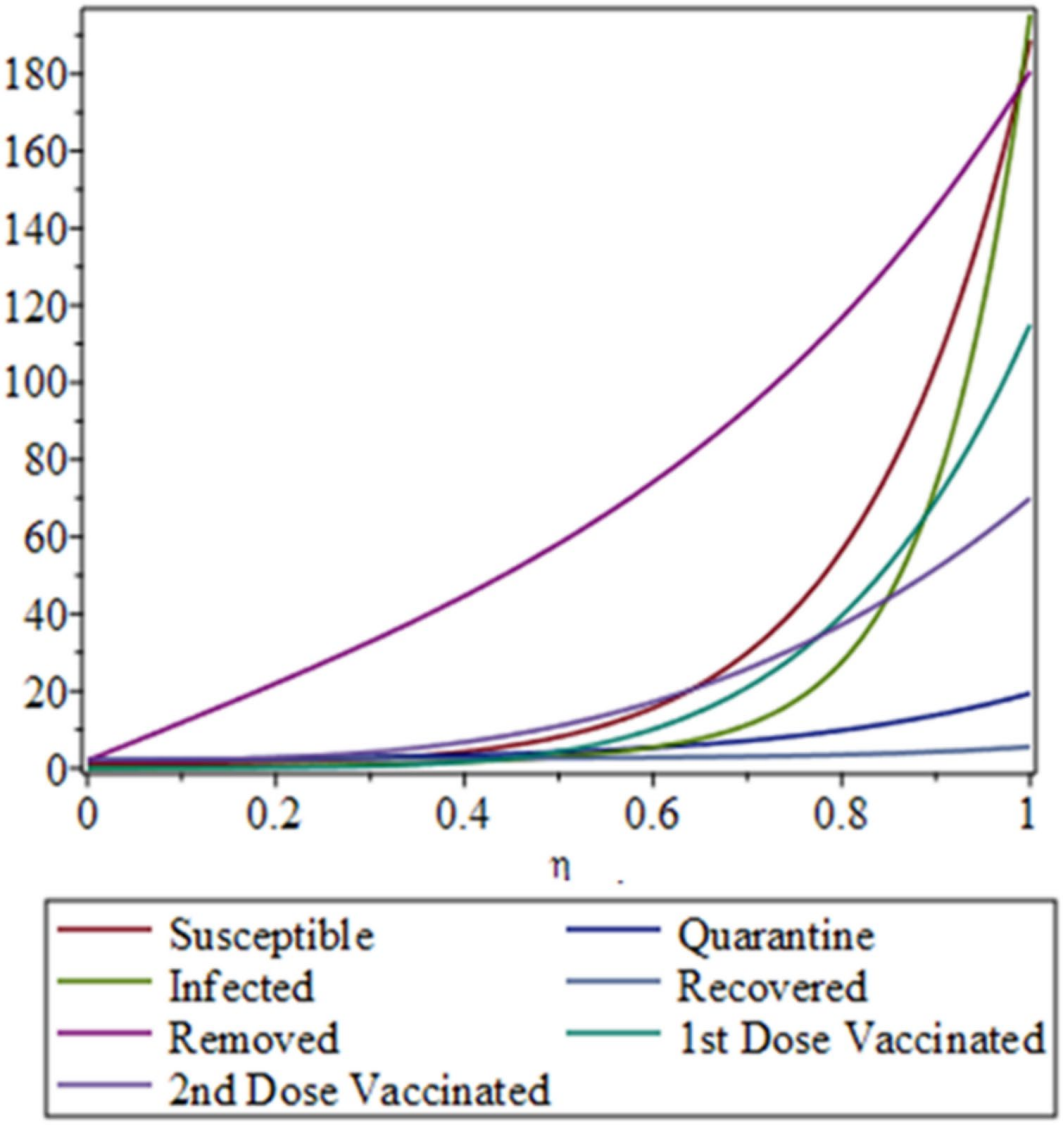
Complete dynamical behavior of the susceptible, infected, quarantined, recovered, and removed populations before vaccination, and after the first and second vaccination doses.

**Figure 5:**
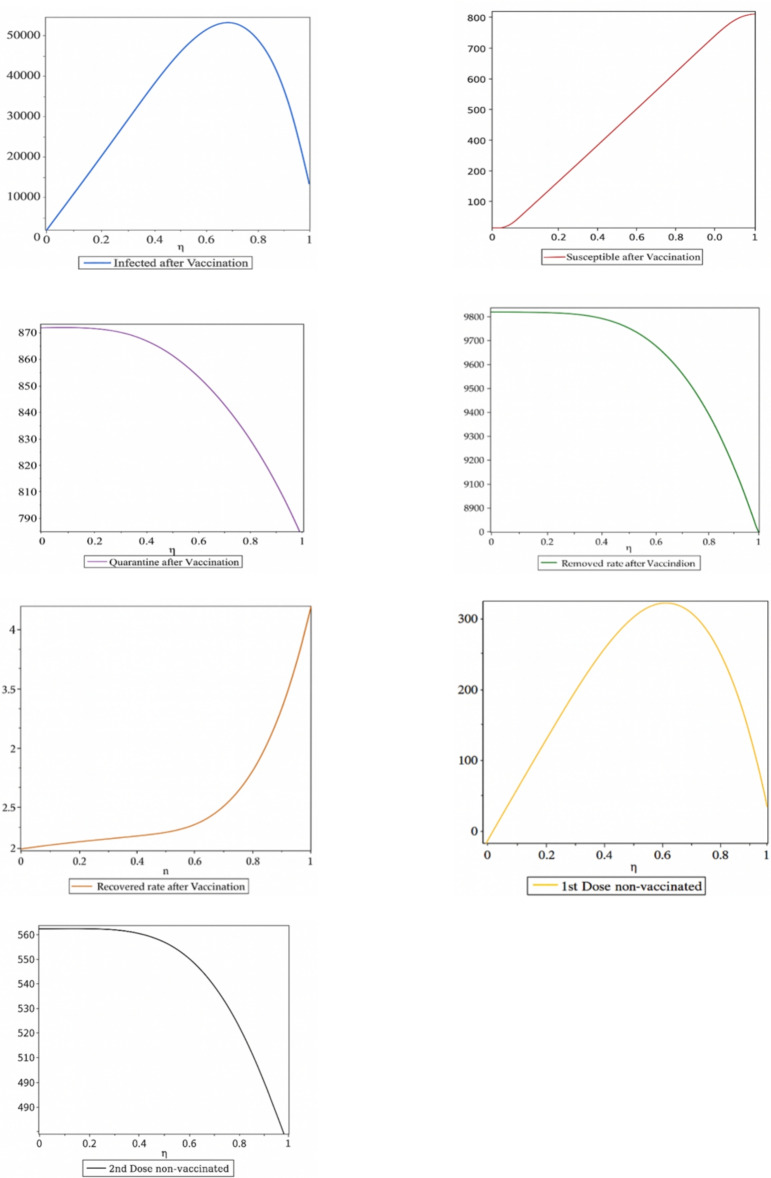
Dynamics of the susceptible, infected, recovered, and removed populations after vaccination, including individuals vaccinated with the first and second doses.

**Figure 6:**
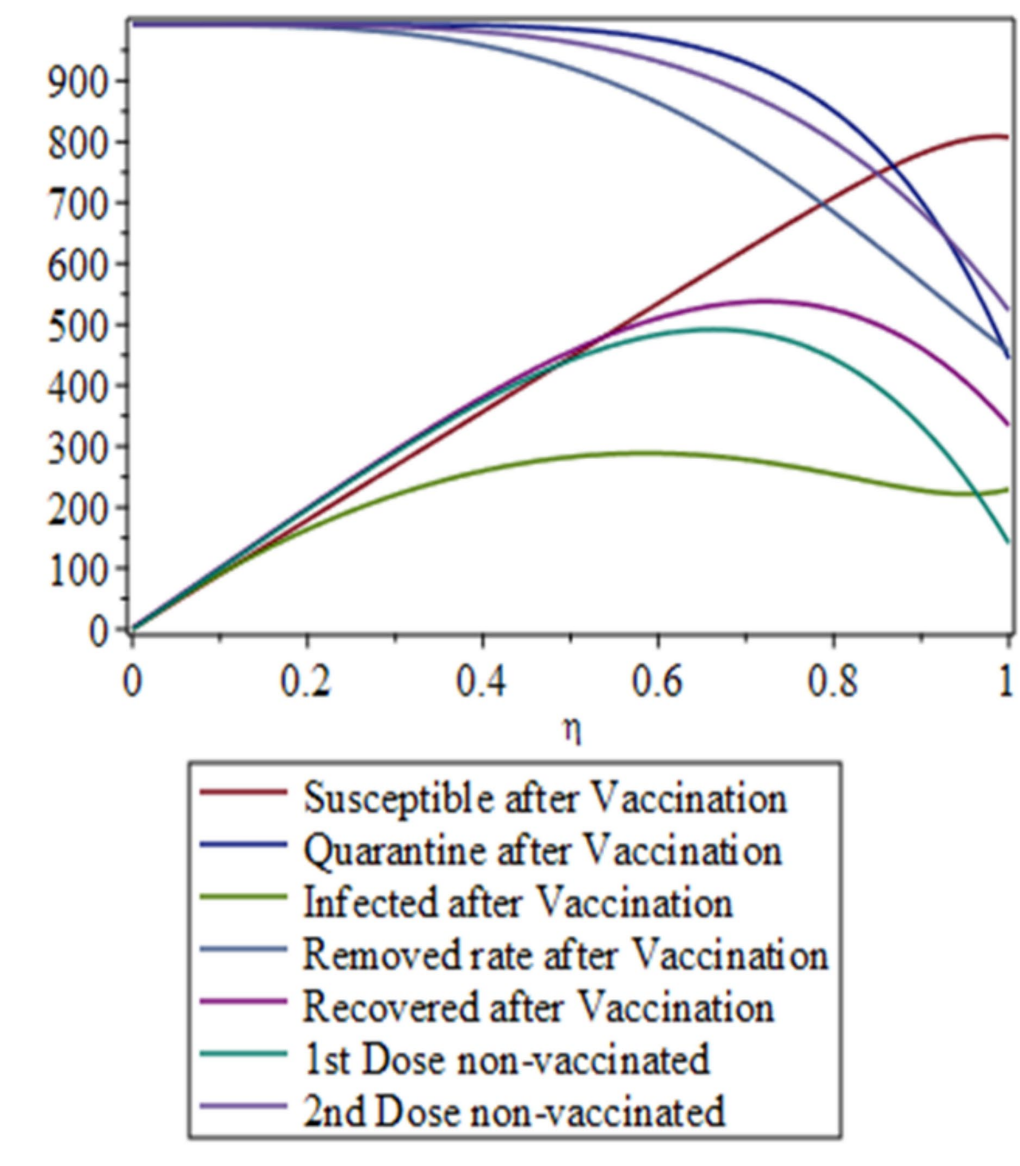
Dynamical analysis of the COVID-19 scenario in Pakistan following the implementation of vaccination.

The dynamical behavior of coronavirus by finding analytical solutions of respective models such as SEIR, SIR and SEQIR has been made easy to understand ^16^. The Variational Iteration Method (VIM) is very operational for studying both linear and non-linear differential equations. But the major objective of the respective method is its ability and flexibility to solve non-linear differential equations accurately and conveniently. Most particularly, this method has the ability to handle linear and non-linear, homogeneous or non-homogenous equations in a better way, and especially reduce the size of calculations ^17–19^. This well-known Variational Iteration Method was first proposed by Ji-Huan He which is a series solution of non-linear differential equations by using an iterative formula. According to Variational Iteration Method, we suppose the stated differential equation

14 $$\begin{aligned} Lu(t) + Nu(t) = g(t), \end{aligned}$$

where *L* is a linear operator, *N* is a non-linear operator and *g*(*t*) is a non-homogeneous source term. We can develop the following correction functional according to Ji-Huan He’s Variational Iteration Method:

15 $$\begin{aligned} u_{n+1}(t) = u_n(t) + \int _0^t \lambda \left( L u_n(\tau ) + N \tilde{u}_n(\tau ) - g(\tau ) \right) d\tau , \end{aligned}$$

where $\lambda$ is the Lagrange multiplier (it may be constant or a function). In this technique, we use integration by parts and restricted variation to determine the value of Lagrange multiplier. We determine the successive approximations $u_n$ of the solution *u* by using the Lagrange multiplier (Figures 3, 4, 5, 6). The zeroth-order approximation $u_0$ can be any selective function. Finally, the solution is:

16 $$\begin{aligned} u(t) = \lim _{n \rightarrow \infty } u_n(t). \end{aligned}$$

### Solution procedure with VIM

The seven non-linear differential equations are given below.

17 $$\begin{aligned} \frac{\textrm{d} S}{\textrm{d} \eta }= \alpha +\varepsilon _{V1}-\beta S-\mu SI-\delta S-\varphi S \end{aligned}$$

18 $$\begin{aligned} \frac{\textrm{d} Q}{\textrm{d} \eta }=\beta S-\kappa Q-\gamma Q-\varphi S \end{aligned}$$

19 $$\begin{aligned} \frac{\textrm{d} I}{\textrm{d} \eta }=\mu SI+\gamma Q-\sigma I-\varphi I \end{aligned}$$

20 $$\begin{aligned} \frac{\textrm{d} R}{\textrm{d} \eta }=\sigma I+\rho _{V2}-\varphi R \end{aligned}$$

21 $$\begin{aligned} \frac{\textrm{d} _{R\circ }}{\textrm{d} \eta }=\varphi S+\varphi Q+\varphi R+\varphi _{V1}+\varphi _{V2}+\omega I \end{aligned}$$

22 $$\begin{aligned} \frac{\textrm{d} _{V1}}{\textrm{d} \eta }=\eta S+\kappa S-\varepsilon _{V1}-\varphi _{V1}-\psi _{V1} \end{aligned}$$

23 $$\begin{aligned} \frac{\textrm{d} _{V2}}{\textrm{d} \eta }=\psi _{V1}-\rho _{V2}-\varphi _{V2} \end{aligned}$$

We select the initial guess as follows for the application of Variational Iteration Method:

24 $$\begin{aligned} _{S0 }\left( \eta \right) =546 _{Q0 }\left( \eta \right) =13 _{I0 }\left( \eta \right) =28 _{R0}\left( \eta \right) =3 _{R\circ 0}\left( \eta \right) =0 V_1^{(0)}(\eta ) = \eta V_2^{(0)}(\eta ) = 1 \end{aligned}$$

According to VIM, the corrections functional are as follows:

25 $$\begin{aligned} S_{n+1}(\eta ) = S_n(\eta ) + \frac{\eta }{4} \left[ S_n(t) - \alpha - \varepsilon V_{1n}(t) + \beta S_n(t) + \mu S_n(t) + \delta S_n(t) + \varphi S_n(t) \right] dt \end{aligned}$$

26 $$\begin{aligned} Q_{n+1}(\eta ) = Q_n(\eta ) + \frac{\eta }{4} \left[ Q_n(t) - \beta S_n(t) + \kappa Q_n(t) + \gamma Q_n(t) + \varphi S_n(t) \right] dt \end{aligned}$$

27 $$\begin{aligned} I_{n+1}(\eta ) = I_n(\eta ) + \frac{\eta }{4} \left[ I_n(t) - \mu S_n(t) I_n(t) + \gamma Q_n(t) + \sigma I_n(t) + \alpha I_n(t) \right] dt \end{aligned}$$

28 $$\begin{aligned} R_{n+1}(\eta ) = R_n(\eta ) + \frac{\eta }{4} \left[ R_n(t) - \sigma I_n(t) - \rho V_{2n}(t) + \varphi R_n(t) \right] dt \end{aligned}$$

29 $$\begin{aligned} R_{\circ _{n+1}(\eta ) = R_{\infty _n}(\eta ) + \frac{\eta }{4} \left[ R_{\infty _n}(t) - \sigma S_n(t) - \varphi Q_n(t) - \varphi R_n(t) - \varphi V_{1n}(t) - \varphi V_{2n}(t) - \alpha I_n(t) \right] dt } \end{aligned}$$

30 $$\begin{aligned} V_{1_{n+1}}(\eta ) = V_{1_n}(\eta ) + \frac{\eta }{4} \left[ V_{1_n}(t) - \delta S_n(t) - \kappa Q_n(t) + \varepsilon V_{1_n}(t) + \varphi V_{1_n}(t) + \psi V_{1_n}(t) \right] dt \end{aligned}$$

31 $$\begin{aligned} V_{2_{n+1}}(\eta ) = V_{2_n}(\eta ) + \frac{\eta }{4} \left[ V_{2_n}(t) - \psi V_{1_n}(t) + \rho V_{2_n}(t) + \varphi V_{2_n}(t) \right] dt \end{aligned}$$

The Lagrange’s Multipliers that we have obtained are as follows:

$$\lambda _1 = -1, \; \lambda _2 = -1, \; \lambda _3 = -1, \; \lambda _4 = -1, \; \lambda _5 = -1, \; \lambda _6 = -1 \; \text {and} \; \lambda _7 = -1$$

32 $$\begin{aligned} S_1(\eta ) = 1 + (1.7000000009 - 3.0600000000 \eta ) \eta \end{aligned}$$

33 $$\begin{aligned} Q_1(\eta ) = 2 + (1.67721565 + k) \eta \end{aligned}$$

34 $$\begin{aligned} _{I1}\left( \eta \right) =\left( 1.32278435-3.01000000\eta -0.500000000\eta \sigma \right) \eta \end{aligned}$$

35 $$\begin{aligned} R_1(\eta ) = 2 + (0.500000000\eta + k + 0.100000000)\eta \end{aligned}$$

36 $$\begin{aligned} R_{\circ _1}(\eta ) = 2 + (0.300000000 + 0.050000000\eta + 0.500000000\eta \omega )\eta \end{aligned}$$

37 $$\begin{aligned} V_{1_1}(\eta ) = (2 - 0.100000000\eta - 0.500000000\eta \psi - k)\eta \end{aligned}$$

38 $$\begin{aligned} V_{2_1}(\eta ) = 2 + (0.100000000 + 0.500000000\eta \psi + k)\eta \end{aligned}$$

39 $$\begin{aligned} {\begin{matrix} S_2(\eta ) & = 1 - 11.08956240\,\eta + 1.84212000\,\eta ^2 \\ & \quad + 1.609266302\,\eta ^3 + 1.27925000\,\eta ^4 \\ & \quad + 0.3069417052\,\eta ^5 + 0.0166666666\,\eta ^6 \\ & \quad + 1.003333333\,\eta \,\sigma - 2.041580848 \\ & \quad + 0.0500000000\,k\,\eta ^2 \end{matrix}} \end{aligned}$$

40 $$\begin{aligned} {\begin{matrix} Q_2(\eta ) & = 1.645568670 + 11.08956240\,\eta + 1.84212000\,\eta ^2 \\ & \quad + 1.609266302\,\eta ^3 + 1.27925000\,\eta ^4 \\ & \quad + 0.8368439356\,\eta ^5 + 2.006666666\,\eta ^6 \\ & \quad + 0.0166666666\,\eta ^7 - 7.567214165\,\eta \\ & \quad - 1.1613921680\,\eta \,\eta \end{matrix}} \end{aligned}$$

41 $$\begin{aligned} {\begin{matrix} I_2(\eta ) & = 1 - \bigl (11.08956240\,\eta + 1.84212000\,\eta ^2 \\ & \quad + 1.609266302\,\eta ^3 + 1.27925000\,\eta ^4 \\ & \quad + 0.3069417052\,\eta ^5 + 0.0166666666\,\eta ^6 \\ & \quad + 1.003333333\,\eta \,\sigma - 2.041580848 \\ & \quad + 0.0500000000\,k\bigr )\eta ^2 \end{matrix}} \end{aligned}$$

42 $$\begin{aligned} R_{\circ _2}(\eta )&= 2 - \bigl (1.003333333\,\eta ^2\,\sigma + 0.1666666666\,\eta ^2 \nonumber \\&\quad - 0.1666666666\,\eta ^7\,k\,\psi - 0.1613921675\,\eta \,\sigma \nonumber \\&\quad - 0.050000000\,\eta \,\kappa - 0.50000000\,\eta \,\kappa ^2 \nonumber \\&\quad - k\bigr )\eta \end{aligned}$$

43 $$\begin{aligned} \begin{aligned} R_2(\eta )&= 2 + (0.200000000 + 0.0166666666\eta ^{2}\sigma + 0.0986666666\eta ^{2} \\&\quad - 1.003333333\eta ^{2}\omega - 0.1666666666\eta ^{2}\omega \sigma + 0.140000000\eta \\&\quad + 0.1613921675\eta \psi + 0.050000000\eta \kappa + 0.0050000000\eta )\eta \end{aligned} \end{aligned}$$

44 $$\begin{aligned} \begin{aligned} V_{1_2}(\eta )&= (3 + 0.0066666666\eta ^{2} + 0.0666666666\eta ^{2}\psi \\&\quad + 0.20000000\eta ^{2}\kappa + 0.1666666666\eta ^{2}\psi ^{2} - 0.300000000\eta \\&\quad - 1.500000000\eta \psi - 0.7500000000\eta \kappa + 0.500000000\eta \kappa \psi - 2k)\eta \end{aligned} \end{aligned}$$

45 $$\begin{aligned} \begin{aligned} V_{2_2}(\eta )&= 2 + (0.166666666\eta ^{2}\kappa \psi - 0.0166666666\eta ^{2}\psi \\&\quad - 0.1666666666\eta ^{2}\psi ^{2} + 0.500000000\eta \psi - 0.500000000\eta \kappa \psi \\&\quad + 0.0050000000\eta + 0.100000000\eta \kappa + 0.500000000\eta \kappa ^{2} \\&\quad + 0.100000000 + \kappa )\eta \end{aligned} \end{aligned}$$

## Numerical predictions and verification

In this section, numerical simulations are performed to validate the proposed mathematical model for the COVID-19 dynamics across Pakistan. The data used in the simulations have been collected from the official website of the National Command and Operation Centre (NCOC) of Pakistan. The values of various parameters employed in the model are estimated and presented in Table 2.

**Table 2:** Values of the parameters used in the mathematical dynamics model for Pakistan.

**Constraints**	**Values**	**Reference**
$\omega$	$98 \times 10^{4}$	Assumed
$\delta$	$0.1 \times 10^{-3}$	Estimated
$\sigma$	$0.5 \times 10^{-4}$	Estimated
$\kappa$	$9.4 \times 10^{-2}$	Estimated
$\psi$	$0.7 \times 10^{-5}$	Estimated
*P*	$3.2 \times 10^{3}$	Estimated
$\epsilon$	$0.6 \times 10^{-3}$	Estimated

## Results and discussion

In this research, we analyse the public health situation in Pakistan from March 2020 onwards using numerical results and graphical simulations. Initially, no effective treatment or vaccine was available, and public awareness about the virus was limited. Many citizens did not take quarantine measures seriously. The collected and plotted data reveal a low recovery rate, primarily due to the absence of treatment and insufficient awareness of the virus’s dangers. After the introduction of vaccines and increased public awareness of their benefits, the proportion of individuals receiving the first dose rose rapidly. The second dose coverage increased more gradually, owing to the recommended 28-day interval between doses. We define the vaccination-related parameter as $\eta = t / T$, where *t* represents time in days and *T* denotes the virus transmission timescale before and after vaccination. Figures 6–7 illustrate the dynamical behaviour of COVID-19 in Pakistan following the vaccination campaign. These figures show that the susceptible and recovered compartments were not significantly affected post-vaccination, as the vaccine primarily provides protection rather than treatment for those already infected. In contrast, the infected and removed compartments exhibit clear declines, reflecting widespread vaccination coverage. The graphs also demonstrate a steady decrease in the proportions of individuals who remained unvaccinated after the first and second doses, indicating that the objectives set by the National Command and Operation Center (NCOC) were successfully met. Our mathematical analysis further reveals a reduction in the quarantine rate, which aligns with the gradual lifting of restrictions across the country. Consequently, economic, educational, and social activities have largely returned to normal. Overall, the results suggest that the disease is approaching eradication in Pakistan.

## Conclusion

In this work, the dynamics of the COVID-19 model have been analysed using the Variational Iteration Method. The disease-free equilibrium and its local stability have been investigated in detail. Additionally, the model has been represented through a signal flow graph (Mason graph), which effectively illustrates the simultaneous population flows among the different compartments. Our mathematical analysis and graphical simulations clearly show that the infection and removal rates were significantly high prior to the introduction of vaccination. The development of COVID-19 vaccines represents a landmark achievement in medical history. The results successfully demonstrate that the infection rate, removal rate, and quarantine rate all declined following the vaccination campaign. Furthermore, this study indicates that the disease has been brought under control–and is approaching eradication–in Pakistan as the proportion of non-vaccinated individuals decreased over time.

## Data Availability

All data used in this study were obtained from the National Command and Operation Centre (NCOC) of Pakistan. These datasets are publicly available and open access through the official NCOC platform. The data contains aggregated COVID-19 statistics and includes no personally identifiable information.
